# Age and geographic heterogeneity in COVID-19 outcomes among young children and parental practices in China

**DOI:** 10.3389/fpubh.2025.1696218

**Published:** 2026-01-12

**Authors:** Beilei Zang, Rongfang Gu, Jinyan Yu, Peng Xu

**Affiliations:** School of Education Sciences, Nanjing Normal University, Nanjing, China

**Keywords:** COVID-19 infection, child health, pediatric infections, education, family health

## Abstract

**Objectives:**

This study examines age and geographic disparities in COVID-19 outcomes among Chinese children aged 0–6 years during China's rapid policy transition (December 2022–January 2023), and evaluates the protective role of parental caregiving practices.

**Methods:**

A cross-sectional online survey leveraging a natural experiment during China's COVID-19 policy shift. Data were collected from 48,332 households through randomized national sampling, stratified by urban/rural and North/South residency. Chi-square tests (SPSS 22.0) and latent class analysis (Mplus 7.0) assessed disparities in infection status, symptom severity, healthcare access, and medication use, alongside parental response patterns.

**Results:**

Age-mediated disparities: Significant gradients in infection rates (χ^2^ = 30.060, *p* < 0.001), symptom severity (χ^2^ = 20.626, *p* < 0.001), and healthcare access (χ^2^ = 90.876, *p* < 0.001), with children aged 3–6 experiencing milder outcomes than infants (0–1 years) and toddlers (1–3 years). Geospatial inequities: Urban-rural gaps in infections (χ^2^ = 58.713, *p* < 0.001) and North-South disparities in medication use (χ^2^ = 71.160, *p* < 0.001), with urban northern children most vulnerable. Parental buffering effects: Parents have exhibited a notably proactive stance and practices in response to the COVID-19 pandemic.

**Conclusions:**

COVID-19 infections in children vary by age and region, with parents playing a crucial role in home-based care. Policy responses should prioritize (1) parent-led protection programs and (2) targeted medical resource allocation to high-risk regions to ensure equitable pandemic recovery.

## 1 Introduction

The COVID-19 pandemic precipitated unprecedented disruptions to global child health ecosystems, exemplified by school closures across 100+ nations affecting 1.6 billion children [United Nations Educational, Scientific and Cultural Organization (UNESCO), 2021; available at https://en.unesco.org/covid19/educationresponse]. Pediatric susceptibility to SARS-CoV-2 manifests primarily through febrile and respiratory symptoms (1, 2), though children generally experience lower infection rates and milder clinical trajectories than adults (3–5). This attenuated risk profile remains counterbalanced by significant household transmission vulnerabilities, as most pediatric cases originate from familial clusters (6–9).

Emerging scholarship reveals that childhood vulnerability, while biologically moderated (10, 11), is fundamentally structured through systemic inequities in healthcare access and geographic disadvantage (12, 13). Critical knowledge deficits persist in three interconnected domains: Clinical-site bias inherent in hospital-centric surveillance obscures community transmission dynamics of mild/asymptomatic pediatric infections (4, 14); structural blindness neglects regional health infrastructure gradients (notably China's North-South divergence) in pediatric outcomes; and household invisibility overlooks intra-familial caregiving mechanisms despite their epidemiological significance (9).

China's pivot from Zero-COVID to endemic management (December 2022-January 2023) constitutes a pivotal natural experiment. This transition enables examination of developmental susceptibility differentials across early childhood stages (0–3 vs. 3–6 years), spatial syndemics reflecting geographic stratification (North/South healthcare divides and urban/rural resource disparities), and caregiving capital through which parental practices potentially buffer structural vulnerabilities.

Heterogeneity in pediatric COVID-19 outcomes is expected, driven by intersecting biological, behavioral, and structural factors. Immunologically, the rapid waning of maternal antibodies and ongoing immune maturation in early childhood create an age-dependent vulnerability gradient (15). Behaviorally, divergent parental caregiving practices across regions mediate exposure risks and healthcare access (16). Geographically, pre-existing disparities in China's healthcare infrastructure and documented North-South differentials in population characteristics establish the preconditions for spatial inequities (17). This study therefore aims to quantify age-stratified and geographically-determined disparities in COVID-19 outcomes among Chinese children aged 0–6 years during this policy transition, while evaluating how parental caregiving practices mediate structural risks. Through nationally representative household-level data (*n* = 48,332), we investigate the reconfiguration of child health vulnerability architectures under rapid epidemiological phase shifts. All participant data were anonymized and stored on secure, encrypted servers to ensure confidentiality, adhering to strict data protection protocols and principles of secure information transmission in digital health studies (18, 19). The primary contributions of this work lie in identifying the most vulnerable subgroups and empirically demonstrating the buffering effect of parental practices, thereby providing timely evidence to inform equitable resource allocation and targeted public health interventions. Based on the aforementioned research inquiries and in conjunction with the literature review, this study posits the following four research hypotheses:

H1: COVID-19 infections in children aged 0–6 manifest milder symptoms.

H2: COVID-19 infections in children aged 0–6 exhibit variations based on age groups.

H3: COVID-19 infections in children aged 0–6 vary across regions.

H4: Parents can effectively respond to COVID-19 infections in children aged 0–6.

## 2 Materials and methods

### 2.1 Sample

This survey is limited to children aged 0–6, with parents serving as respondents. Among the respondents, 77.05% are mothers of the children, 20.36% are fathers, and the remaining respondents are other family members, such as grandparents. The survey encompasses provinces across mainland China, including 22 provinces, five autonomous regions, and four municipalities directly under the central government, totaling 31 provinces. Geographic classification was based on the official standard used by China's National Bureau of Statistics. Given that the pandemic control measures in Hong Kong, Macao, and Taiwan differed from those in Mainland China, these regions were not included in the current survey.

We developed an online questionnaire on the official website of Wenjuanxing (www.wjx.cn), a recognized professional platform for online surveys, assessments, and polls. During the Chinese New Year period in January 2023, we conducted a nationwide survey of parents of children aged 0–6 regarding the infection status of this age group with the novel coronavirus. We collected 48,357 responses through Wenjuanxing. After excluding 25 invalid responses, we obtained a total of 48,332 valid responses.

The process for obtaining electronic informed consent was as follows: Before accessing the survey questions, all potential participants were directed to a dedicated information page. This page comprehensively outlined the research objectives, the estimated time commitment, potential risks and benefits, and the procedures for ensuring data anonymity and confidentiality. It was explicitly stated that participation was entirely voluntary, that participants had the right to withdraw at any time without penalty, and that by proceeding to the questionnaire, they were providing their consent. To proceed, participants were required to actively indicate their agreement by selecting a checkbox next to the statement: “I have read and understood the above information, and I voluntarily agree to participate in this study.” Only upon selecting this option could they advance to the actual survey. This digital opt-in procedure served as the equivalent of written informed consent in the online survey context and was a mandatory step for all respondents.

In our cross-sectional study of caregivers of children aged 0–6 in China, we employed a hybrid sampling method combining a panel-based approach with social media/snowball sampling. To address the potential selection bias inherent in this method, we implemented a *post-hoc* re-stratification procedure. This ensured that key variables—child's age, geographic region, and urban-rural classification—were balanced across groups in our comparative subgroup analyses.

### 2.2 Questionnaire

The questionnaire comprises three sections, totaling 16 questions. The first part covers demographic information, the second part investigates the infection status of children aged 0–6 years with the novel coronavirus, and the third part primarily explores parental practices and recommendations. Parental caregiving practices were assessed via a self-reported online questionnaire, developed from literature on outbreak health behaviors and Chinese public health guidelines. It measured multiple dimensions—preventive behaviors, environmental management, and illness management—with respondents reporting the frequency of or adherence to these practices over a specified recall period. Since this survey consists of non-scale questions, the researcher primarily ensures the credibility and reliability of the questionnaire data through processes such as data collection, ensuring the validity of the sample, and handling invalid samples. From the perspective of content validity, the questionnaire's reasonableness and effectiveness are ensured. Through communication and discussion with multiple experts and others, and based on rigorous analysis, adjustments were made to the questionnaire survey questions, ultimately forming the final version of the questionnaire for this study.

### 2.3 Data analysis

Data analysis was conducted using SPSS 22.0 and Mplus 7.0. SPSS was employed for descriptive statistics and frequency analysis of demographic characteristics and COVID-19 related information, and chi-square tests were utilized to analyze differences in the infection status of children. Additionally, Mplus was employed for analyzing the latent features of children's COVID-19 infections.

## 3 Results

### 3.1 Overall status of COVID-19 infection in children aged 0–6

Nationwide data revealed 73.64% of households experienced concurrent adult-child (0–6 years) COVID-19 infections, vs. 11.94% adult-only infections. Children predominantly contracted infection after adults (56.47%, *p* < 0.05). Symptom severity comparisons showed 71.27% of children exhibited milder manifestations than adults, significantly exceeding those with more severe symptoms (3.78%, *p* < 0.05). Clinically, 54.83% of infected children presented attenuated cough symptoms (*p* < 0.05), while 49.15% developed moderate fevers (38.5–40 °C, *p* < 0.05). Healthcare utilization data indicated 61.58% required no hospitalization, significantly surpassing both necessary (*p* < 0.05) and actual care-seeking proportions.

Collectively, pediatric infections demonstrated four cardinal characteristics: delayed onset relative to adult household members, attenuated symptom severity vs. adults, prevalent moderate fever presentation, and reduced cough salience.

### 3.2 Analysis of age and regional differences in COVID-19 infection among children aged 0–6

#### 3.2.1 Comparison of COVID-19 infection status in children of different age groups

Analysis of 1,551 children per age group (0–1, 1–3, 3–6 years) revealed significant COVID-19 outcome disparities (all *p* < 0.001; cough homogeneity excluded). Infection rates differed (χ^2^ = 30.060, *p* = 0.000): toddlers peaked (81.11%) > infants (76.02%) > preschoolers (72.86%). Preschoolers showed mildest symptoms (87.60%; χ^2^ = 20.626, *p* = 0.000) vs. toddlers (82.43%) and infants (81.50%).

Fever patterns diverged (χ^2^ = 144.479, *p* = 0.000): toddlers had highest moderate fever (38.5–40 °C: 57.00%), infants more low-grade fever (< 38.5 °C: 27.85%), preschoolers highest afebrile cases (32.69%). Medication use varied (χ^2^ = 30.932, *p* = 0.000): infants favored non-pharmaceutical patches (22.00%), toddlers repeated antipyretics (60.43% twice), preschoolers single doses (28.83% once).

Healthcare utilization demonstrated inverse age gradients (χ^2^ = 90.876, *p* = 0.000): infants showed highest foregone care (22.17%) and outpatient visits (14.35%); preschoolers lowest care avoidance (10.80%) and highest no need (79.98%). Medical intervention requirements declined with age.

#### 3.2.2 Comparison of COVID-19 infection status in children in different regions

Comparative analysis of pediatric COVID-19 outcomes across Chinese regions revealed differential viral strains (Northern BF.7 vs. Southern BA.5.2; CCTV Network, 2022; available at https://m.gmw.cn/baijia/2022-12/13/1303223199.html). To understand whether there are differences in the infection status of children in northern and southern regions, the researchers selected data from Beijing and Guangdong for comparison. Beijing-Guangdong comparisons showed homogeneity in infection timing, adult-relative symptom severity, cough manifestations, and peer transmission rates (all *p* > 0.05), with both regions exhibiting delayed adult-to-child transmission, attenuated pediatric symptoms, and elevated peer infection.

Significant interregional disparities emerged: Beijing children demonstrated higher infection rates (χ^2^ = 5.822, *p* = 0.016), increased twice-daily antipyretic use (χ^2^ = 71.160, *p* < 0.001, Cramer's V = 0.632), and greater medical treatment needs (χ^2^ = 7.169, *p* = 0.028, Cramer's V = 0.183) vs. Guangdong counterparts.

Urban-rural stratification revealed substantial infection differentials (χ^2^ = 58.713, *p* < 0.001). Concurrent adult-child infections predominated in urban households (77.30%), exceeding rural (72.90%) and town residences (67.75%). Symptom severity and clinical manifestations showed no significant settlement-type variation.

#### 3.2.3 Exploring age and regional characteristics of children's infection status based on latent class analysis

Latent class analysis (LCA) of 24,602 children aged 0–6 years examined five COVID-19 severity dimensions: symptom intensity, fever occurrence, medication use, cough manifestations, and healthcare-seeking. The analysis was guided by several goodness-of-fit indices, including the Akaike Information Criterion (AIC), Bayesian Information Criterion (BIC), and Adjusted BIC (ABIC). To determine the optimal number of latent classes, the researchers considered the values of these indices alongside the Entropy measure and performed statistical tests such as the Lo-Mendell-Rubin Likelihood Ratio Test (LMR) and Bootstrapped Likelihood Ratio Test (BLRT). Significance in the *p*-values associated with LMR and BLRT indicated that a model with K classes was superior to a model with K-1 classes (20). Following a thorough comparison of the numerical results derived from the latent profile analysis, the researchers opted for a model comprising two distinct classes, as outlined in Table 1.

**Table 1:** Fit indices for latent class analysis model selection.

**Model**	***K***	**Log (L)**	**AIC**	**BIC**	**ABIC**	**Entropy**	**LMR *p***	**BLRT *p***	**Latent class probability**
1	10	−114,116.703	228,253.405	228,334.511	228,302.731				
2	16	−96,695.793	193,423.586	193,553.355	193,502.507	1.000	0.000	0.000	0.865/0.135
3	22	−91,936.216	183,916.433	184,094.866	184,024.950	0.876	0.000	0.000	0.291/0.574/0.135

Class distribution revealed C1 (“Low Severity”: 86.52%) and C2 (“High Severity”: 13.48%). Chi-square analysis confirmed significant inter-class differences by region (χ^2^ = 9.196, *p* = 0.010) and age (χ^2^ = 8.587, *p* = 0.014; Table 2).

**Table 2:** Distribution of geographical area and age groups across latent classes.

**Demographic characteristics**	**Profile 1 *N* = 21,285**	**Profile 2 *N* = 3,317**	**χ^2^**
**Geographical area**			
Cities	54.84%	56.53%	9.196^*^
Towns	23.15%	20.77%	
Villages	22.01%	22.70%	
**Age groups**			
0–12 months	2.96%	3.75%	8.587^*^
13–36 months	5.14%	5.72%	
3–6 years	91.90%	90.53%	

^*^p < 0.05.

**Figure 1:**
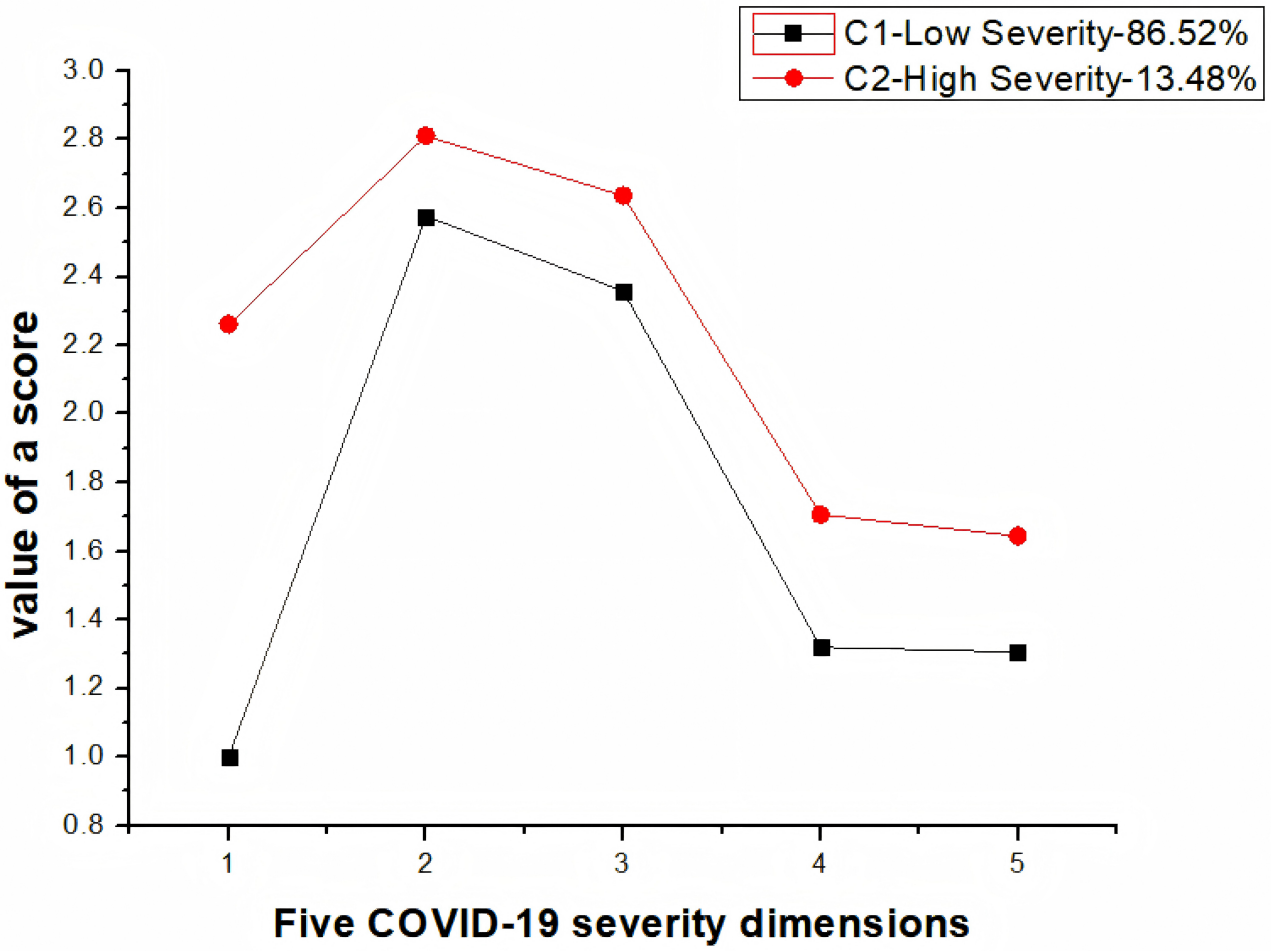
Category mean plot of COVID-19 infection status in children.

As Figure 1 illustrates, C1 demonstrated systematically lower severity across all indicators. This profile predominated among preschoolers (3–6 years: 91.90%) and town residents (23.15%). Conversely, C2 exhibited elevated clinical severity across all five domains.

### 3.3 Parental attitudes and practices toward COVID-19

In regards to the survey of parents, four relevant questions were included, covering parents' actions, awareness of protection, preventive measures, and advice regarding children's new coronavirus infection.

In response to the COVID-19 pandemic, 77.59% of parents attempted to provide their children with rudimentary explanations based on their own understanding. The number of parents attempting to explain was significantly higher across all age groups compared to those who did not attempt to explain or did not explain. There are age differences in parents' explanations to children (χ^2^ = 40.0888, *p* = 0.000). The proportion of parents of 3–6-year-old children who “provided a superficial explanation based on their understanding” (84.38%) was significantly higher than the other two age groups. Regarding whether children need protective masks, parents of all age groups have a high percentage of agreement on the need for mask-wearing, and there is no significant difference between age groups (χ^2^ = 5.947, *p* = 0.203).

The research also found that 61.23% of parents believe that “frequent hand-washing and maintaining cleanliness” is the most important daily measure to prevent infectious diseases, 24.99% of parents chose “increasing exercise and ensuring nutrition,” and 11.55% of parents believe that “providing necessary health education to children” is essential. Furthermore, parents' suggestions regarding how to prevent the invasion of the new coronavirus in 0–6-year-old children primarily focus on frequent hand-washing (28.82%), mask-wearing (25.82%), hygiene awareness (19.62%), increased exercise (10.19%), improved nutrition (8.29%), and better ventilation (7.03%).

## 4 Discussion

Leveraging China's policy transition as a natural experiment, this study reveals how structural factors—age gradients, geographic stratification, and parental practices—intersect to shape COVID-19 outcomes among young children. Our data were collected during January 2023, which coincided with the Chinese New Year holiday and the initial, massive infection wave following the cessation of the Dynamic Zero-COVID policy. Our findings must be interpreted within the unique context of China's Dynamic Zero-COVID policy, wherein state-mandated measures such as mass testing, and centralized quarantine acted as massive confounding variables, simultaneously shaping viral transmission pathways, healthcare access, and household-level behaviors. Our findings advance theoretical understanding of child health vulnerability during public health crises and offer actionable insights for equitable pandemic response.

### 4.1 Age-mediated vulnerability: a dual lens of biological and social constructs

Our research findings confirm that children aged 0–6 infected with COVID-19 typically exhibit milder symptoms compared to adults, as evidenced by lower proportions of high fever, severe cough, and hospitalization needs. This aligns with previous global studies (1, 21), reinforcing the understanding that children demonstrate a distinct response to the virus. However, from a social science perspective, this age-mediated vulnerability is not solely a biological phenomenon but is deeply intertwined with social structures and practices.

On the one hand, the relatively low exposure risk among young children is significantly influenced by social care arrangements. In Chinese society, children aged 0–6 often receive intensive care at home, with limited opportunities for independent social interactions. This caregiving pattern, shaped by cultural norms and family structures, was further intensified by the policy context, effectively reduces their direct contact with the pathogen and infected individuals. On the other hand, the unique immune response in children, which may contribute to milder symptoms, is also affected by social determinants. For instance, access to healthcare and vaccination programs, which are integral parts of the social welfare system, can impact the development and function of children's immune systems. The potential role of the MMR vaccine in preventing COVID-19 in children underscores the importance of public health policies and vaccination coverage, both of which are influenced by social and political decisions (22–24).This highlights how social factors interact with biological mechanisms to shape the vulnerability of young children to COVID-19, emphasizing the need for a comprehensive understanding that transcends biological reductionism.

### 4.2 Geospatial stratification: structural inequities in infection landscapes

Our study reveals intricate age and regional variations in COVID-19 infections among children aged 0–6 in China, indicating that vulnerability is not uniformly distributed but is stratified by multiple social factors. Preschool children (3–6 years) showed the lowest infection rate and milder symptoms, while toddlers (1–3 years) had the highest infection rate with more severe symptoms and longer fever durations. Infants (0–1 year) occupied an intermediate position but had the highest proportion of seeking medical care. These age-related disparities can be partly attributed to differences in social interactions and caregiving practices. Preschool children, who are more likely to be in organized educational settings with established health protocols, may have benefited from collective preventive measures. In contrast, toddlers, with their increased mobility and exploratory behavior, may be more exposed to the virus in home and community environments.

Geospatial stratification further exacerbates these disparities. Urban children had a relatively higher infection rate but milder infection conditions compared to rural children, reflecting differences in access to healthcare resources, living environments, and social support systems. Moreover, the implementation intensity of Dynamic Zero-COVID measures (e.g., frequency of mass testing, strictness of mobility controls) likely varied between urban and rural areas, as well as between northern and southern regions, creating a heterogeneous policy landscape that contributed to the observed infection patterns. Urban areas, with more advanced medical facilities and public health infrastructure, may be better equipped to diagnose and manage mild infections promptly, contributing to less severe outcomes. When comparing northern and southern regions, children in Beijing had higher infection rates, medication frequencies, and medical care-seeking proportions than those in Guangdong. These regional differences are likely influenced by a combination of factors, including climate, population density, local policy enforcement, and the effectiveness of local public health policies. Such findings underscore the significance of considering social and spatial inequalities in formulating targeted public health interventions. Child healthcare departments and institutions need to adopt region- and age-specific strategies, strengthening prevention and control measures in high-risk areas and age groups. Moreover, promoting equitable access to healthcare resources across urban and rural areas and regions is crucial for reducing health disparities among young children during and after pandemics.

### 4.3 Parental involvement: a structural buffer against vulnerability

Our research highlights that Chinese parents demonstrated a proactive stance in response to the COVID-19 pandemic, yet there was a notable gap between their beliefs and actual practices in child protection. While 95% of parents recognized the importance of children wearing masks for protection, only a small proportion of children actually wore masks in practice. This discrepancy reflects the complex interplay between parental intentions and the practical challenges of implementing preventive measures for young children. Children's resistance due to high activity levels or breathing discomfort, combined with the difficulty of enforcing mask-wearing in daily life, underscores the need for more creative and child-friendly preventive strategies.

Despite this gap, parents' adoption of other preventive measures, such as regular handwashing, demonstrates their ability and willingness to protect their children. Handwashing, an effective preventive method supported by research (8), became the top choice for parents in both daily prevention and recommendations. This indicates that when practical and feasible preventive measures are available, parents are more likely to implement them successfully. The high reported adherence to hygiene practices may also reflect the heightened public health messaging and behavioral norms established during the prolonged Zero-COVID campaign. Moreover, parents' attention and care for children at risk of contracting COVID-19, as well as their efforts to explain the virus to their children based on their understanding, highlight the crucial role of parental practices in buffering the impacts of the pandemic on young children. However, the indirect impacts of the COVID-19 pandemic and public health measures on children, including psychological effects, should not be overlooked (10, 25–27). Chinese parents' attempts to communicate with their children about the virus, which increased with children's age, suggest that parental education and communication play an important role in helping children cope with the pandemic. Future research and public health interventions should focus on providing parents with more practical guidance and support to bridge the gap between intentions and practices, enhance the effectiveness of preventive measures, and address the comprehensive needs of children during public health crises.

## 5 Limitations and future research directions

Despite its contributions, this study has several notable limitations. First, the cross-sectional nature of our survey limits the ability to infer causality in the relationships between structural factors, parental practices, and child health outcomes. While this design was justified for capturing a unique, rapid policy transition, future research should employ longitudinal or quasi-experimental designs to establish causal pathways and unravel the complex interplay between structural inequities and behavioral adaptations over time.

Second, the uneven distribution of valid questionnaires across different regions in mainland China may introduce sampling bias, potentially skewing the statistical results and affecting the accuracy of regional comparisons. This imbalance reflects broader social and economic disparities in survey participation, which could influence the representativeness of the findings for certain subpopulations. Third, the study's results are situated within the specific COVID-19 policies and socioeconomic conditions of mainland China. The unique social, political, and cultural context may limit the generalizability of the findings to other countries or regions. Fourth, while this study identified differential distributions of symptom severity classes across geographic and age groups, it did not formally test for statistical interactions between parental practices and demographic factors (e.g., whether the protective effect of mask-wearing varied by residential area) using moderated regression models. Finally, caregiving practices were self-reported and thus susceptible to social desirability bias, likely inflating the reported prevalence of behaviors like mask-wearing. Therefore, these figures represent upper-bound estimates, and their absolute levels should be interpreted with caution. However, this bias is unlikely to invalidate the relative associations we observed across groups, unless the bias itself differed systematically between them.

Building on these findings and limitations, future research should pursue several directions. To better differentiate structural from behavioral mechanisms, mixed-methods studies are urgently needed to qualitatively explore the decision-making processes of parents in different structural contexts. Furthermore, future research should incorporate interaction analyses to better understand how the effectiveness of specific caregiving behaviors may be contingent upon contextual factors. Additionally, technological advancements offer promising tools. Research could leverage artificial intelligence (AI) to model viral evolution and predict the long-term public health impacts of regional strain variations. This aligns with the promising field of the Intelligent Internet of Medical Things (IIoMT), which offers a pathway for real-time, low-cost monitoring of child health in underserved regions, as evidenced by its application in mental health (28–30). The methodological approaches used in related studies, such as machine learning to classify parental perspectives (31), could also be adopted to refine risk prediction models for young children.

## 6 Conclusion

COVID-19 infections among children aged 0–6, especially those aged 3–6, generally manifest with milder symptoms compared to adults. There exist age and regional variations in COVID-19 infections among children aged 0–6. Parental practices buffer risks but face implementation limits. Prioritizing parent-led protection and targeted medical resource allocation to high-risk regions is critical for equitable pandemic resilience.

## Data Availability

The original contributions presented in the study are included in the article/supplementary material, further inquiries can be directed to the corresponding authors.
